# Molecular Dynamics Simulations of Displacement Cascades in BCC-Fe: Effects of Dislocation, Dislocation Loop and Grain Boundary

**DOI:** 10.3390/ma16237497

**Published:** 2023-12-04

**Authors:** Pandong Lin, Shugang Cui, Junfeng Nie, Lei He, Wendong Cui

**Affiliations:** 1 Key Laboratory for Liquid-Solid Structural Evolution and Processing of Materials, Ministry of Education, Shandong University, Jinan 250061, China; 2 School of Materials Science and Engineering, Shandong University, Jinan 250061, China; 3 Key Laboratory of Advanced Reactor Engineering and Safety, Ministry of Education, Institute of Nuclear and New Energy Technology, Tsinghua University, Beijing 100084, China

**Keywords:** molecular dynamics, displacement cascade, dislocation, dislocation loop, grain boundary

## Abstract

The interactions between displacement cascades and three types of structures, dislocations, dislocation loops and grain boundaries, in BCC-Fe are investigated through molecular dynamics simulations. Wigner–Seitz analysis is used to calculate the number of point defects induced in order to illustrate the effects of three special structures on the displacement cascade. The displacement cascades in systems interacting with all three types of structure tend to generate more total defects compared to bulk Fe. The surviving number of point defects in the grain boundary case is the largest of the three types of structures. The changes in the atomic structures of dislocations, dislocation loops and grain boundaries after displacement cascades are analyzed to understand how irradiation damage affects them. These results could reveal irradiation damage at the microscale. Varied defect production numbers and efficiencies are investigated, which could be used as the input parameters for higher scale simulation.

## 1. Introduction

In nuclear power plants, many structural materials such as A508-III steel and tungsten are exposed to large amounts of neutron irradiation, which could result in irradiation hardening, embrittlement and creep [1,2,3]. For example, irradiation results in increased hardness, yield stress and ductile–brittle transition temperature. Molecular dynamics (MD) simulation has been extensively used to study defect production from displacement cascades in bulk body-centered cubic (BCC), face-centered cubic (FCC) and close-packed hexagonal (HCP) metals [4,5,6,7,8,9,10]. For example, Sahi et al. [7] conducted molecular dynamics simulations to investigate the effect of applied strain and temperature on a displacement cascade in Zr. Their results demonstrated that the number of defects induced by the displacement cascade increased under tensile uniaxial hydrostatic strain. Many irradiation defects are generated during the displacement cascade process. Results show that the number of point defects after a displacement cascade depends on varied factors such as energy of primary knocked-on atoms (PKA) and temperature.

As the microstructure evolves, the dislocations, dislocation loops and grain boundaries could become the dominant defects in materials [11,12]. Indeed, dislocations and grain boundaries are inherent defects in materials while dislocation loops are attributed to irradiation. Therefore, the three types are the typical structures when considering both irradiated and unirradiated factors. Apart from this, displacement cascade processes may interact with these defects, leading to varied defect production numbers and efficiencies, which could be used as the input parameters for higher scale simulations such as dislocation dynamics (DD) simulation and crystal plasticity (CP) theory. For example, Roman. E [13] studied the collision cascade process when interacting with 1/2<110> screw dislocations in aluminum. The number of residual point defects was evaluated and compared with that in the pristine material under the same irradiation conditions. Dai et al. [14] used molecular dynamics simulations to reveal that the primary damage production was reduced due to the presence of *a*-type dislocation loops, *c*-component dislocation loops and a tilt grain boundary. Fu et al. [15] found that the residual vacancy number usually exceeded the residual interstitial number when the displacement cascades interacted with straight edge dislocation in tungsten via a molecular dynamics simulation. However, the influence of these defects on the displacement cascade in BCC-Fe is not extensively studied, especially the understanding of surviving defects induced by the displacement cascade. In addition, a comparison of varied interactions due to different structures also needs to be studied. The purposes are (1) clarifying the interaction mechanism between the defect structure and the displacement cascade and (2) obtaining varied defect production numbers and efficiencies, in order to provide input parameters for different structures in the mesoscale model.

In addition, MD simulations suggest that the point defects induced via the displacement cascade may enter the structure of dislocation segments or grain boundaries. For example, S. Heredia-Avalos et al. [16] found that a migration of the 1/2<111> edge dislocation by gliding along its slip plane could occur, which was attributed to a collision cascade through an atomic simulation. Fu et al. [15] found that the cascade near a dislocation promoted climb and some of dipoles were sheared off by the cascade. Lu et al. [17] stated that the dislocation climb in edge dislocations and cross-slip in screw dislocations are caused by displacement cascades in Fe-20Cr-25Ni alloys, observed through molecular dynamics. It could be concluded that these structures are changed deeply in the presence of a displacement cascade. Note that clarifying the influence of the displacement cascade process on defect structures is also of concern in this work. At present, there are few comparison studies on the interaction between the three types of defect structures and displacement cascades.

In this study, we investigate the interactions between displacement cascades and three types of structures, dislocations, dislocation loops and grain boundaries, using MD simulations in BCC-Fe, which is basic components of structural materials [18]. The effects of three types of structures on the total and surviving defect number are determined. The atomic structural changes in dislocations, dislocation loops and grain boundaries induced by displacement cascades are analyzed.

## 2. Methods

Large-scale atomic/molecular massively parallel simulator (LAMMPS) code [19,20] is used to launch all MD simulations. The simulation system contains around 3.3 million atoms, as shown in Table 1, and periodic boundary conditions are applied in all three directions. The interatomic potentials calculated using the embedded atom method (EAM) developed by Mendelev et al. [21] were applied to describe the inter-atomic Fe interactions, and were successfully applied to simulate irradiation damage in BCC Fe [22,23,24].

### 2.1. Generation of Edge Dislocation, Screw Dislocation, Dislocation Loop and Grain Boundary

**Figure 1:**
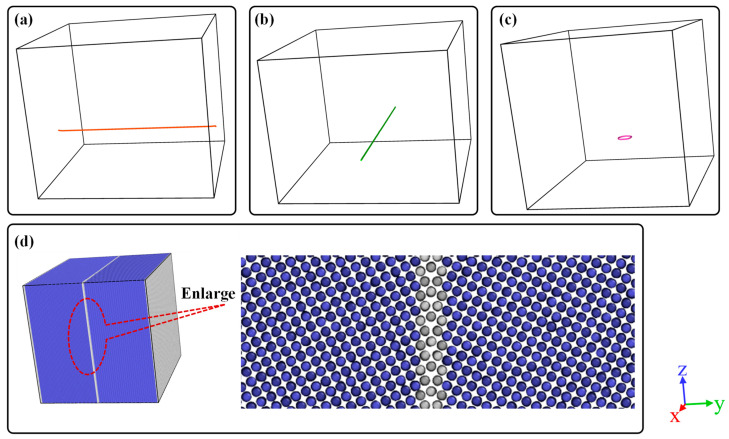
Schematic of defects in a simulation box: (**a**) 1/2[1–11] screw dislocation, (**b**) 1/2[1–11] edge dislocation, (**c**) [001] dislocation loop and (**d**) ∑5(013) grain boundary. BLue balls represent atoms in bcc structure while white balls denote atoms of grain boundary.

Before the displacement cascade simulation, the accurate models of edge dislocation, screw dislocation, the dislocation loop and the grain boundary should be introduced, respectively. 1/2[1–11] edge dislocation with the x slip direction and screw dislocation with the y slip direction were established using Atomsk software (version beta-0.12.1) [25]. The BCC crystal containing dislocation was oriented along the x [112], y [11–1] and z [−110] directions. When it comes to the dislocation loop, the model consisting of a [001] dislocation loop whose diameter is 3.5 nm was oriented along the x [100], y [010] and z [001] directions. Moreover, the habit plane of the interstitial-type dislocation loop is perpendicular to their Burgers vectors. Note that the [001] dislocation loop is square-shaped. The ∑5(013) grain boundary was also built using Atomsk. Figure 1d shows the configuration of the ∑5(013) grain boundary where the tilt axis is aligned along the x direction, and the grain boundary planes are normal to the y direction. The schematic of simulation cells is shown in Figure 1 and the details of the simulation cells used in this work are listed in Table 1. Furthermore, the conjugate gradient method was applied to all cases to determine the global minimum energy configurations. Such being the case, the relaxed configuration could be obtained.

### 2.2. Displacement Cascade Simulations

The schematic of the displacement cascade simulation box is shown in Figure 1. The PKA energy was 10 keV and the temperature was set as 300 K. The PKA energy is given in the form of atomic velocity and the PKA was placed close to the dislocation, dislocation loop and grain boundary within 3.0 nm with the aim of investigating the interaction between cascade events and special structures. For each case, 8 independent cases were simulated with random seeds. In all cases, it should be guaranteed that the displacement cascades evolved and relaxed for several picoseconds until the temperature and the generated number of defect displacements stabilized. Note that the constant-energy constant-volume ensemble (NVE) was used in all cascade simulations. Before initial displacement damage simulations, all simulation cells of Fe crystals were relaxed for 50 ps in the NPT ensemble to ensure that the system reached thermal equilibrium. The cell dimensions were held constant for further displacement cascade simulations. An adaptive time step method [19,20] is used during displacement cascade simulations to ensure the appropriate displacement of atoms per time step. Here, the maximum distance for an atom to move in one timestep is 0.00125 nm.

### 2.3. Analysis and Visualization Tools

Common Neighbor Analysis (CNA) [26,27] is used to distinguish the structure of atoms (BCC, FCC and HCP and so on). The number of interstitials and vacancies is counted using the Wigner–Seitz analysis [28]. A vacancy is represented by an empty W-S cell with no Fe atoms, whereas a W-S cell with two Fe atoms corresponds to an interstitial. The dislocation extraction method (DXA) [29] is used to examine the dislocation and its Burgers vector. All analyses were completed using the Open Visualization Tool (OVITO) [30] code.

### 2.4. Defect Counting Method

Below is described the defect analysis method. At first, Wigner–Seitz analysis was used to calculate the total number of interstitials (I_t_) and vacancies (V_t_). Then, it could be found that some portion of the point defects (interstitials or vacancies) moved into dislocations, dislocation loops and grain boundaries. This portion of the point defects are defined as I_d_ and V_d_. The other part resides in the matrix of material, which are named as I_m_ and V_m_. Generally, it could be obtained that I_t_ = I_d_ + I_m_ and V_t_ = V_d_ + V_m_.

## 3. Results and Discussion

### 3.1. Cascade Simulation of BCC-Fe with Dislocations

In this section, the cascade simulations of BCC-Fe with 1/2<111> edge and screw dislocations are analyzed from two perspectives. The first part is the defect production. The other part is the displacement-cascade-induced atomic structural changes in the edge and screw dislocations, respectively.

#### 3.1.1. The Influence of Dislocations on Defect Production

**Figure 2:**
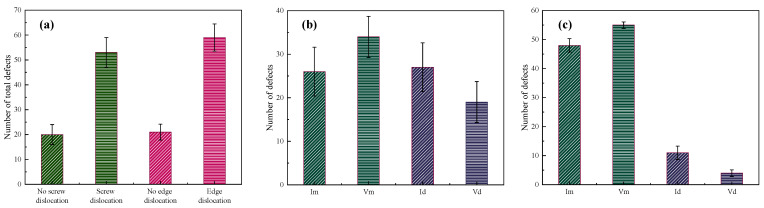
The number of point defects induced by displacement cascade for BCC-Fe with or without the dislocations: (**a**) The effect of screw and edge dislocation on the total number of point defects. (**b**) The effects of screw dislocation on I_m_, V_m_, I_d_, V_d_. (**c**) The effects of edge dislocation on I_m_, V_m_, I_d_, V_d_.

Figure 2 represents the number of point defects induced by the displacement cascade for BCC-Fe. The simulated data points are averaged from the eight samples generated from the random seeds, which is used in Section 3.2 and Section 3.3. The standard deviation is also listed in Figure 2. It could be seen that displacement cascade tends to increase the number of total defects when interacting with both screw and edge dislocations as shown in Figure 2a. Whether edge dislocation or screw dislocation, there are more than twice as many point defects in the presence of the dislocation as in the absence of a dislocation. As has been mentioned in Section 2, the point defects may go into dislocations or be still left in the materials after the displacement cascade. If we focus on the surviving point defects left in the matrix, the number of defects (I_m_ and V_m_ in Figure 2b,c) for the cases of both edge and screw dislocations is more than that in the case without dislocation, which means that the defect production efficiency in the materials with high dislocation density is higher. Apart from that, it could be concluded that (a) more interstitials as opposed to vacancies move into dislocation for both screw and edge dislocations, which could suppress the migration of interstitials and their recombination with vacancies, leading to more point defects left in the simulation system, and (b) compared with edge dislocations, screw dislocations are more likely to absorb point defects. More discussions about structural changes are listed in the next part.

#### 3.1.2. Atomic Structural Changes in Dislocations

**Figure 3:**
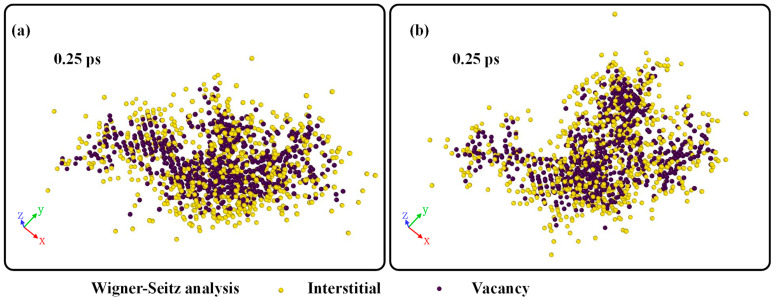
The distribution of interstitials and vacancies during displacement cascade simulation: (**a**) Edge dislocation and (**b**) Screw dislocation.

Figure 3 shows the distribution of interstitials and vacancies during the displacement cascade simulation at 0.25 ps. It could be seen that the interstitials basically surround the vacancies for the cases with both edge and screw dislocations. High-energy particles knock the struck atom off its lattice site, creating a vacancy. The dislocated atoms continue to emit outwards, hitting other lattice atoms; such being the case, the interstitials are at the periphery of the displacement cascade region.

**Figure 4:**
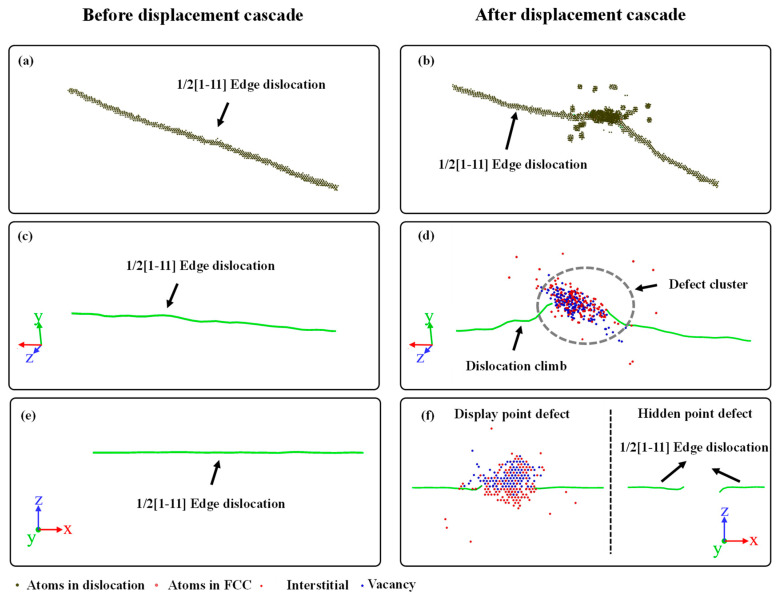
The atomic structure of an edge dislocation. (**a**,**c**,**e**) are the structures before displacement cascade. (**b**,**d**,**f**) are the structures after displacement cascade.

**Figure 5:**
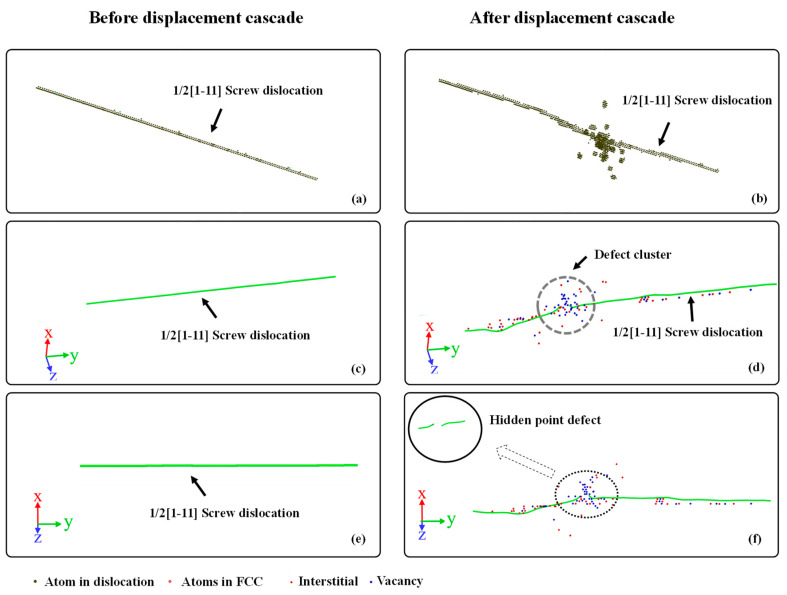
The atomic structure of a screw dislocation. (**a**,**c**,**e**) are the structures before displacement cascade. (**b**,**d**,**f**) are the structures after displacement cascade.

Figure 4 and Figure 5 show the atomic structures of the edge and screw dislocations before and after the displacement cascade for BCC-Fe, using the CNA and DXA analysis. The Burgers vector of both the edge and screw dislocations is set as 1/2[1–11].

For the edge dislocation, the following could be concluded: (1) The migration and bending of dislocation along the y direction are clearly discernible. In terms of dislocation under a fixed temperature, no motion of dislocation occurs. Therefore, the motion of dislocation during the displacement cascade is attributed to the temperature gradient. Apart from that, with the arrival of the irradiation-induced point defects, dislocation segments in the displacement cascade region actually climb, leading to the bending of the dislocation. (2) On the other hand, the displacement cascade would eliminate some atoms of dislocation as shown in Figure 4f. Generally, the extra-high energy is introduced by the PKA, as a consequence of which some atoms of the dislocation that are in the displacement cascade region become amorphous during the displacement cascade. The dislocation is divided into two small dislocation segments when it cools down. (3) The displacement cascade could facilitate dislocation climb, which can be seen in Figure 4d,f. Note that dislocation climb could be divided into negative and positive [17]. The preference for positive or negative dislocation climb depends on the type of defects that go into dislocations. For a positive climb to happen, the edge dislocation needs to absorb vacancies, while the edge dislocation needs to absorb interstitials to have a negative climb. In other words, the availability of interstitials and vacancies determines whether a negative climb or positive climb occurs. (4) A point defect cluster is generated in the dislocation core, as shown in Figure 4d. Except for a few interstitial atoms and vacancy-entry dislocations, most point defects exist in the form of clusters.

For screw dislocations, there are many similarities with edge dislocations. It can be seen that some of the atoms are also eliminated by the cascade as shown in Figure 5f. Moreover, a point defect cluster also forms due to the interaction between the screw dislocation and the cascade, as shown in Figure 5d. In contrast to the dislocation climb of edge dislocations, the screw dislocation may cross-slip during the interaction. Furthermore, note that the number of point defects entering the screw dislocation is significantly higher than that entering the edge dislocation, which explains the fewer surviving interstitials and vacancies in the matrix of BCC-Fe. Last but not least, two small dislocation segments are also observed in the finial atomic configuration.

### 3.2. Cascade Simulation of BCC-Fe with Dislocation Loop

**Figure 6:**
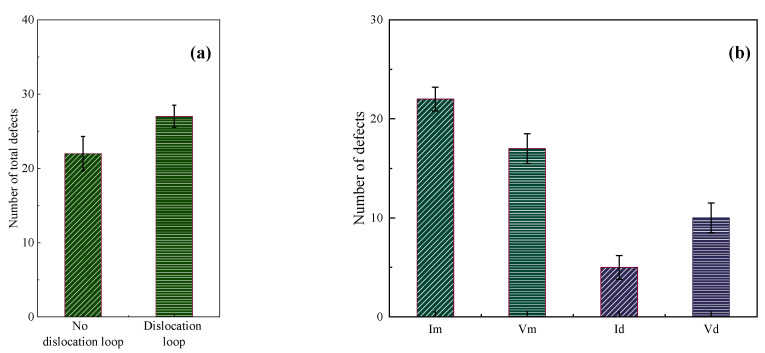
The number of point defects induced by the displacement cascade for BCC-Fe with or without the dislocation loops: (**a**) The effect of [001] dislocation loop on the total number of irradiation-induced point defects. (**b**) The effects of [001] dislocation loop on I_m_, V_m_, I_d_, V_d_.

In this section, the effect of the [001] dislocation loop on the displacement cascade simulation of BCC-Fe is discussed. Figure 6 shows the number of point defects induced by the displacement cascade for BCC-Fe with or without the dislocation loops. It could be concluded that (1) more total point defects are generated when displacement cascades interact with the [001] dislocation loop, as shown in Figure 6a, and (2) fewer surviving defects are left in the matrix of BCC-Fe when compared to the case without a dislocation loop. Such being the case, the defect production efficiency in the BCC-Fe with a high density of [001] dislocation loops is lower than in the bulk case.

**Figure 7:**
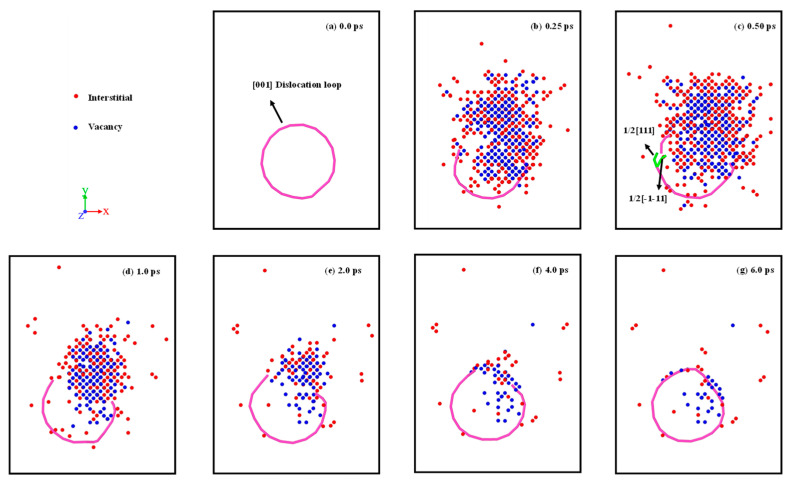
The interaction evolution between dislocation loop and displacement cascade.

Figure 7 shows the interaction evolution between the dislocation loop and the displacement cascade. The following could be found: (1) At first, the cascade eliminates some of the atoms in the dislocation loop as shown in Figure 7b, which is similar to the case of 1/2<111> dislocation. (2) By interacting with the displacement cascade, dislocation dissociation occurs, which can be described as [001] → 1/2[111] + 1/2[−1–11], as shown in Figure 7c. Due to the instability of the 1/2[111] and 1/2 [−1–11] dislocation segments, dislocation dissociation disappears in the interaction with the displacement cascade. (3) In the later part of the displacement cascade process, the [001] dislocation segment is regenerated due to the recovery of interstitials and vacancies, and finally a complete [001] dislocation loop is formed. Note that the interstitials and vacancies induced by the displacement cascade enter the dislocation loop, which can be seen in Figure 7e. Unlike the 1/2<111> dislocation loop, the <100> dislocation segment is more likely to adsorb vacancies rather than interstitials.

### 3.3. Cascade Simulation of BCC-Fe with Grain Boundary

**Figure 8:**
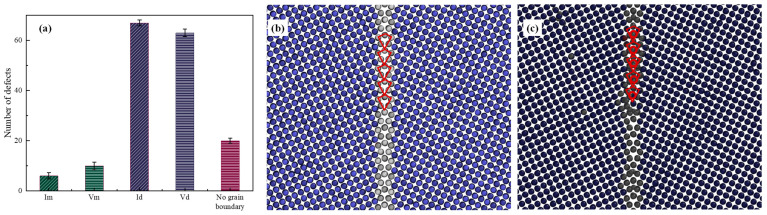
(**a**) The effects of ∑5(013) grain boundary on I_m_, V_m_, I_d_ and V_d_. Projection from 〈100〉 axis on GBs, showing the change in structures: (**b**) the grain boundary is ‘kite’-shaped without the cascade and (**c**) the grain boundary remains ‘kite’-shaped with the cascade. Bule balls represent atoms in bcc structure while white balls denote atoms of grain boundary.

In this part, the displacement cascade interacting with the grain boundary is investigated. Figure 8 shows the effects of the ∑5(013) grain boundary on I_m_, V_m_, I_d_ and V_d_. It can be seen that a displacement cascade that overlaps a grain boundary feeds the grain boundary with excess defects, leaving few defects in the matrix of BCC-Fe. In other words, the ∑5(013) grain boundary could reduce the surviving interstitials in bulk Fe by 70% and vacancies by 55%, and from this the grain boundary could be seen as having weak biased sinks for the annihilation of irradiation-induced defects. This is echoed by the fact that the grain boundary could trap defects but preferentially trap interstitials [31,32,33]. This phenomenon has also been observed in W [34], Mo [35], Fe-Cr alloys [36] and Fe [37]. Moreover, the time for stabilization of the irradiation damage in the grain boundary system is longer than in bulk Fe, which is attributed to the preferential sink property of the grain boundary over point defects. In terms of the structure of the grain boundary, the initial structure is ‘kite’-shaped [38], as shown in Figure 8b. When interacting with the displacement cascade, the trapped interstitials fill the largest free volume within the grain boundary, retaining the ‘kite’ structure, which is consistent with previous work [39]. The difference lies in the fact that the ‘kite’ shape has an extra atom in its center, as shown in Figure 8c. Moreover, the reversible grain boundary phase transition, reported in FCC metals [38], is not found in this work.

**Figure 9:**
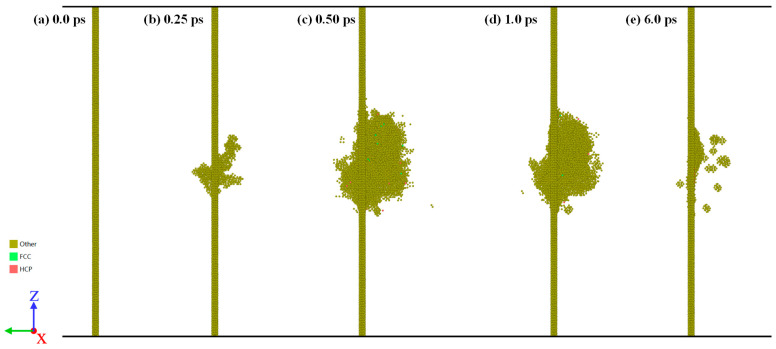
Evolution of the displacement cascade with the simulation time in the ∑5(013) grain boundary. The atoms of BCC structure are hidden, as identified via the CNA method.

Figure 9 shows the evolution of the displacement cascade with the simulation time in the presence of the ∑5(013) grain boundary. Note that the grain boundaries seem to migrate slightly towards the side of the incoming PKA, which means the displacement cascade could damage the grain boundary structure. Furthermore, this could be attributed to both the temperature gradient during the cascade evolution as well as the excess point defect produced in the displacement cascade [39].

## 4. Conclusions

In this work, a molecular dynamics method is used to investigate the interaction between a displacement cascade and three types of structures: dislocations, dislocation loops and grain boundaries. The defect production and the displacement cascade induced atomic structural changes are discussed briefly. Below are listed the conclusions.

- Due to the presence of dislocations, dislocation loops and grain boundaries, the defect production efficiency changes. All three types of structures can cause the cascade to generate more total point defects. Moreover, a portion of these defects enter into the dislocation, dislocation loop and grain boundary. During the interaction, 1/2[1–11] edge dislocation climb occurs while screw dislocation may cross-slip. Compared with edge dislocations, screw dislocations are more likely to absorb point defects.
- As for the [001] dislocation loop, a lower number of surviving defects is left in the matrix of BCC-Fe, which means that the defect production efficiency in the BCC-Fe with a high density of [001] dislocation loops is lower than in the bulk case. Compared to the 1/2<111> dislocation segment, the <100> dislocation segment is more likely to adsorb vacancies rather than interstitials.
- The number of point defects in the grain boundary case is the largest of the three types of structures. Grain boundaries could trap defects but preferentially trap interstitials. Furthermore, grain boundaries seem to migrate slightly towards the side of the incoming PKA, but retain their ‘kite’ structure.

This paper explores the effects of three types of defects: dislocations, dislocation loops and grain boundaries, on the displacement cascade process, which can reveal irradiation damage at the microscale. Varied defect production numbers and efficiencies are investigated, which could be used as the input parameters for higher scale simulations. Future research can be carried out based on parameter transformation between microscale and higher scale simulations.

## Figures and Tables

**Table 1:** Type of defects and size and atoms in the simulation cells.

Structure	x	y	z	Number of Atoms
BCC 1/2[1–11] edge dislocation	35.0 nm	37.2 nm	30.3 nm	3,390,600
BCC 1/2[1–11] screw dislocation	32.0 nm	37.1 nm	32.3 nm	3,233,622
BCC [001] dislocation loop	34.3 nm	34.3 nm	34.3 nm	3,456,177
BCC ∑5(013) grain boundary	27.1 nm	27.1 nm	27.1 nm	1,710,000

## Data Availability

All the data that support the findings of this study are available upon reasonable request.
